# Draft Genome Sequences of 158 Listeria monocytogenes Strains Isolated from Black Bears (Ursus americanus) in the United States

**DOI:** 10.1128/mra.00248-23

**Published:** 2023-06-05

**Authors:** Phillip Brown, Yi Chen, Mirena Ivanova, Pimlapas Leekitcharoenphon, Cameron Parsons, Jeffrey Niedermeyer, Nicholas Gould, Jennifer Strules, J. Bernardo Mesa-Cruz, Marcella J. Kelly, Michael J Hooker, Michael J. Chamberlain, Colleen Olfenbuttel, Christopher DePerno, Driss Elhanafi, Sophia Kathariou

**Affiliations:** a Department of Plant and Microbial Biology, North Carolina State University, Raleigh, North Carolina, USA; b Division of Microbiology, Center for Food Safety and Applied Nutrition, Food and Drug Administration, College Park, Maryland, USA; c Research Group for Genomic Epidemiology, National Food Institute, Technical University of Denmark, Lyngby, Denmark; d Department of Food, Bioprocessing and Nutrition Sciences, North Carolina State University, Raleigh, North Carolina, USA; e Fisheries, Wildlife, and Conservation Biology, Department of Forestry and Environmental Resources, North Carolina State University, Raleigh, North Carolina, USA; f Department of Fish and Wildlife Conservation, Virginia Tech, Blacksburg, Virginia, USA; g Integrative Sciences, Harrisburg University, Harrisburg, Pennsylvania, USA; h Warnell School of Forestry and Natural Resources, University of Georgia, Athens, Georgia, USA; i North Carolina Wildlife Resources Commission, Raleigh, North Carolina, USA; j Biomanufacturing Training and Education Center, North Carolina State University, Raleigh, North Carolina, USA; University of Maryland School of Medicine

## Abstract

Listeria monocytogenes is responsible for severe foodborne disease and major economic losses, but its potential reservoirs in natural ecosystems remain poorly understood. Here, we report the draft genome sequences of 158 L. monocytogenes strains isolated from black bears (Ursus americanus) in the southeastern United States between 2014 and 2017.

## ANNOUNCEMENT

Listeria monocytogenes is a facultative intracellular Gram-positive bacterial pathogen responsible for the severe foodborne disease listeriosis (1). Three serotypes (i.e., 1/2a, 1/2b, and 4b) predominate in human listeriosis, with 4b being the leading contributor and including all major hypervirulent clones (1, 2). L. monocytogenes is notorious for its persistence in food processing environments (FPEs), but the actual sources of the strains that colonize FPEs remain poorly understood (3). Food animals and agricultural environments have been extensively investigated, while potential reservoirs in natural ecosystems such as wildlife remain underexplored.

We found that wild black bears (Ursus americanus) in the southeastern United States were frequently colonized by L. monocytogenes without apparent disease symptoms, with 12% of the samples yielding serotype 4b (4). A significant portion of 4b strains exhibited the multiplex PCR profile IVb-vI encountered in emerging clones such as ST382 and 554 (3, 4). Here, we report the whole-genome sequence (WGS) data of 158 L. monocytogenes strains from these black bears, including 84 and 57 of serotype 1/2a and 4b, respectively (Table 1). WGS analysis revealed 68 sequence types (STs) based on the multilocus sequence typing (MLST) scheme. Surprisingly, the generally-ubiquitous STs 1, 2, 4, and 6 (5) were markedly uncommon, with only four strains of ST1 and none of ST2, ST4, or ST6. Lineage III, frequently encountered in nonhuman animals (6), was encountered in only nine strains. Dominant groups were clonal complex 554 (CC554) (serotype 4b; *n* = 28), CC388 (serotype 4b; *n* = 11), and CC20 (serotype 1/2a; *n* = 9). The majority of the STs (42/68, ca. 62%) were novel (Table 1).

**TABLE 1:** Characteristics of the 158 Listeria monocytogenes strains from black bears (Ursus americanus) described in this report

Strain name	Serotype	ST^*a*^	CC	Isolation		Bear		Sample type	No. of contigs	Total length (bp)	GC content (%)	*N*_50_ (bp)	Coverage (×)	No. of reads	Sequencing method	Read quality control and trimming tools	Accession no.			
				Yr	City, state, country	Identifier	Capture coordinates										GenBank	BioSample	Assembly	Sequence Read Archive
SKB39	1/2a	788	7	2014	NC, USA	UNK 1	Unknown	Feces	14	2,886,019	37.9	1,493,457	78	534,583	Illumina MiSeq	CLC Genomics Workbench 7.5.1	AAZCOI000000000	SAMN17815015	GCA_017121895.1	SRR13642977
SKB109	1/2a	900	7	2014	NC, USA	UNK Caswell Orphan	Unknown	Feces	18	2,909,257	37.9	1,481,159	93	665,817	Illumina MiSeq	CLC Genomics Workbench 7.5.1	AAZCLO000000000	SAMN17815130	GCA_017120345.1	SRR13643217
SKB403	1/2a	365	14	2016	Asheville, NC, USA	N056	35.584589 N, −82.621004 W	Nasal swab	67	2,932,209	37.9	428,130	1,170	13,346,689	Illumina NextSeq 500	FastQC v0.11.5, BBDuk v38.89	AAAXDY000000000	SAMN08183176	GCA_004626455.1	SRR6394897
SKB557	1/2a	**1388**	14	2016	Asheville, NC, USA	N131	35.648864 N, −82.479895 W	Feces	30	2,902,622	37.9	339,136	484	4,857,222	Illumina NextSeq 500	FastQC v0.11.5, BBDuk v38.89	AAAXDK000000000	SAMN08183184	GCA_004625815.1	SRR6392039
SKB560	1/2a	**1388**	14	2016	Asheville, NC, USA	N131	35.648864 N, −82.479895 W	Rectal swab	23	2,953,489	37.8	1,504,505	60	461,506	Illumina MiSeq	CLC Genomics Workbench 7.5.1	AAZCRA000000000	SAMN17814197	GCA_017123355.1	SRR13642676
SKB165	1/2a	20	20	2015	Asheville, NC, USA	N061	35.581290 N, −82.518469 W	Rectal swab	18	2,970,085	37.9	525,811	424	4,304,067	Illumina MiSeq	CLC Genomics Workbench 7.5.1	AAZASH000000000	SAMN17815064	GCA_017069515.1	SRR13643076
SKB23	1/2a	20	20	2014	Asheville, NC, USA	N024	35.581683 N, −82.515916 W	Feces	18	2,858,149	37.9	583,919	65	397,990	Illumina MiSeq	CLC Genomics Workbench 7.5.1	AAZCOM000000000	SAMN17814492	GCA_017122155.1	SRR13642960
SKB47	1/2a	20	20	2014	Macon, GA, USA	UGA169	Unknown	Rectal swab	23	3,028,268	37.8	299,142	48	303,351	Illumina MiSeq	CLC Genomics Workbench 7.5.1	AAZCNT000000000	SAMN17815018	GCA_017121245.1	SRR13642985
SKB612	1/2a	20	20	2016	Asheville, NC, USA	N087	35.603350 N, −82.527849 W	Feces	18	3,039,614	37.8	562,688	183	1,915,619	Illumina MiSeq	CLC Genomics Workbench 7.5.1	AAZAGV000000000	SAMN17814371	GCA_017059255.1	SRR13642909
SKB217	1/2a	**1384**	20	2015	Asheville, NC, USA	N043	35.499100 N, −82.550667 W	Rectal swab	15	2,972,380	37.9	525,813	119	1,215,566	Illumina MiSeq	CLC Genomics Workbench 7.5.1	AAZARA000000000	SAMN17815039	GCA_017069215.1	SRR13643125
SKB362	1/2a	**1384**	20	2015	Asheville, NC, USA	N095	35.517192 N, −82.573830 W	Rectal swab	19	2,973,852	37.9	525,813	247	2,582,252	Illumina MiSeq	CLC Genomics Workbench 7.5.1	AAZAQA000000000	SAMN17815131	GCA_017068775.1	SRR13643229
SKB455	1/2a	**1384**	20	2016	Asheville, NC, USA	N116	35.517192 N, −82.573830 W	Nasal swab	21	2,892,383	37.9	583,956	1,170	12,258,689	Illumina NextSeq 500	FastQC v0.11.5, BBDuk v38.89	AAAXEL000000000	SAMN08183153	GCA_004623875.1	SRR6395536
SKB528	1/2a	**1384**	20	2016	Asheville, NC, USA	N125	35.648864 N, −82.479895 W	Feces	22	2,848,312	37.9	583,887	1,027	10,377,382	Illumina NextSeq 500	FastQC v0.11.5, BBDuk v38.89	AAAXEX000000000	SAMN08183182	GCA_004622345.1	SRR6397281
SKB740	1/2a	**1484**	20	2017	Asheville, NC, USA	N151	35.591109 N, −82.538996 W	Rectal swab	16	2,936,976	37.9	573,660	105	1,049,569	Illumina MiSeq	CLC Genomics Workbench 7.5.1	AAZAGY000000000	SAMN17814274	GCA_017059075.1	SRR13642684
SKB690	1/2a	321	321	2016	Asheville, NC, USA	N145	35.684397 N, −82.576257 W	Feces	18	3,000,799	37.8	489,037	190	1,938,787	Illumina MiSeq	CLC Genomics Workbench 7.5.1	AAZAGX000000000	SAMN17814134	GCA_017059375.1	SRR13642651
SKB197	1/2a	**1370**	570	2015	Asheville, NC, USA	N059	35.646382 N, −82.492551 W	Nasal swab	27	2,848,964	37.9	570,292	456	4,617,926	Illumina NextSeq 500	FastQC v0.11.5, BBDuk v38.89	AAAXDZ000000000	SAMN08183171	GCA_004625055.1	SRR6395184
SKB116	1/2a	**1362**	787	2014	Asheville, NC, USA	N049	35.522016 N, −82.477618 W	Rectal swab	12	2,976,860	37.9	544,553	102	721,551	Illumina MiSeq	CLC Genomics Workbench 7.5.1	AAZCPW000000000	SAMN17814358	GCA_017122695.1	SRR13642907
SKB117	1/2a	**1362**	787	2014	Asheville, NC, USA	N049	35.522016 N, −82.477618 W	Rectal swab	11	2,939,966	37.9	526,146	124	860,770	Illumina MiSeq	CLC Genomics Workbench 7.5.1	AAZCQL000000000	SAMN17814323	GCA_017123095.1	SRR13642767
SKB100	1/2a	838	838	2014	Asheville, NC, USA	N040	35.496265 N, −82.503329 W	Swab	126	2,870,294	37.9	43,622	60	596,869	Illumina NextSeq 500	FastQC v0.11.5, BBDuk v38.89	DAKHKH000000000	SAMN31891312	GCA_026470765.1	SRR22428071
SKB137	1/2a	838	838	2014	Asheville, NC, USA	N058	35.652544 N, −82.535214 W	Feces	24	2,891,928	37.9	436,201	70	497,362	Illumina MiSeq	CLC Genomics Workbench 7.5.1	AAZCPD000000000	SAMN17814481	GCA_017122395.1	SRR13642946
SKB140	1/2a	838	838	2014	Asheville, NC, USA	N059	35.637214 N, −82.498245 W	Feces	42	2,875,338	37.9	292,529	35	348,942	Illumina MiSeq	CLC Genomics Workbench 7.5.1	AAZAQQ000000000	SAMN17815088	GCA_017069155.1	SRR13643175
SKB144	1/2a	**1368**	906	2014	VA, USA	VT BBRC 119	Unknown	Rectal swab	16	2,917,963	37.9	525,325	139	957,279	Illumina MiSeq	CLC Genomics Workbench 7.5.1	AAZCQI000000000	SAMN17814324	GCA_017123075.1	SRR13642766
SKB187	1/2a	**1368**	906	2015	VA, USA	VT BBRC 119	Unknown	Nasal swab	22	2,871,559	37.9	524,454	929	10,194,404	Illumina NextSeq 500	FastQC v0.11.5, BBDuk v38.89	AAAXDN000000000	SAMN08183180	GCA_004627565.1	SRR6392095
SKB190	1/2a	**1368**	906	2015	VA, USA	VT BBRC 119	Unknown	Nasal swab	26	2,867,340	37.9	517,587	457	4,564,458	Illumina NextSeq 500	FastQC v0.11.5, BBDuk v38.89	AAAXDN000000000	SAMN08183173	GCA_004625795.1	SRR6396393
SKB427	1/2a	912	912	2016	Asheville, NC, USA	N110	35.584093 N, −82.531423 W	Rectal swab	25	2,933,152	37.8	299,475	1,265	13,648,343	Illumina NextSeq 500	FastQC v0.11.5, BBDuk v38.89	AAAXDJ000000000	SAMN08183175	GCA_004626415.1	SRR6394294
SKB429	1/2a	912	912	2016	Asheville, NC, USA	N110	35.584093 N, −82.531423 W	Swab	56	2,951,097	37.9	147,691	71	718,963	Illumina NextSeq 500	FastQC v0.11.5, BBDuk v38.89	DAKCDO000000000	SAMN31788316	GCA_026352655.1	SRR22336611
SKB397	1/2a	**1383**	912	2016	Asheville, NC, USA	N095	35.517192 N, −82.573830 W	Nasal swab	26	2,961,311	37.8	271,119	772	8,477,955	Illumina NextSeq 500	FastQC v0.11.5, BBDuk v38.89	AAAXDM000000000	SAMN08183169	GCA_004623015.1	SRR6394323
SKB398	1/2a	**1383**	912	2016	Asheville, NC, USA	N095	35.517192 N, −82.573830 W	Nasal swab	34	2,967,471	37.8	186,234	76	774,220	Illumina NextSeq 500	FastQC v0.11.5, BBDuk v38.89	DAKCDN000000000	SAMN31788315	GCA_026352635.1	SRR22336613
SKB593	1/2a	913	913	2016	Asheville, NC, USA	N136	35.635396 N, −82.525301 W	Rectal swab	18	3,054,592	37.8	439,804	245	2,573,081	Illumina MiSeq	CLC Genomics Workbench 7.5.1	AAZAHM000000000	SAMN17814125	GCA_017059535.1	SRR13642643
SKB599	1/2a	920	920	2016	Asheville, NC, USA	N137	35.532643 N, −82.595264 W	Feces	17	3,011,733	37.9	437,579	253	2,627,535	Illumina MiSeq	CLC Genomics Workbench 7.5.1	AAZAGZ000000000	SAMN17814127	GCA_017059395.1	SRR13642645
SKB632	1/2a	920	920	2016	Asheville, NC, USA	N061	35.517192 N, −82.573830 W	Nasal swab	19	2,925,003	37.9	1,478,054	81	637,413	Illumina MiSeq	CLC Genomics Workbench 7.5.1	AAZCRB000000000	SAMN17814180	GCA_017123395.1	SRR13642670
SKB719	1/2a	920	920	2016	Asheville, NC, USA	N138	35.517192 N, −82.573830 W	Nasal swab	18	3,050,496	37.8	437,585	193	2,017,452	Illumina MiSeq	CLC Genomics Workbench 7.5.1	AAZARP000000000	SAMN17815012	GCA_017069475.1	SRR13642975
SKB285	1/2a	935	935	2015	Asheville, NC, USA	N084	35.652551 N, −82.535369 W	Rectal swab	18	2,943,324	37.8	433,614	59	598,692	Illumina MiSeq	CLC Genomics Workbench 7.5.1	AAZARD000000000	SAMN17815069	GCA_017069195.1	SRR13643130
SKB333	1/2a	935	935	2015	Asheville, NC, USA	N015	35.511962 N, −82.529787 W	Rectal swab	17	3,102,371	37.8	498,362	375	3,990,037	Illumina MiSeq	CLC Genomics Workbench 7.5.1	AAZAQO000000000	SAMN17815091	GCA_017068535.1	SRR13643193
SKB339	1/2a	935	935	2015	Asheville, NC, USA	N093	35.619101 N, −82.507734 W	Rectal swab	23	2,967,758	37.9	297,084	716	7,488,147	Illumina NextSeq 500	FastQC v0.11.5, BBDuk v38.89	AAAXEK000000000	SAMN08183158	GCA_004626155.1	SRR6395469
SKB343	1/2a	935	935	2015	Asheville, NC, USA	N093	35.619101 N, −82.507734 W	Nasal swab	14	3,052,216	37.8	526,690	410	4,276,797	Illumina MiSeq	CLC Genomics Workbench 7.5.1	AAZAQM000000000	SAMN17815093	GCA_017068555.1	SRR13643188
SKB473	1/2a	935	935	2016	Asheville, NC, USA	N119	35.592583 N, −82.525923 W	Feces	23	2,972,025	37.9	297,080	914	9,509,848	Illumina NextSeq 500	FastQC v0.11.5, BBDuk v38.89	AAAXDU000000000	SAMN08183151	GCA_004622335.1	SRR6394749
SKB751	1/2a	935	935	2017	Asheville, NC, USA	N156	35.600941 N, −82.537208 W	Feces	15	2,993,701	37.8	511,924	155	1,575,853	Illumina MiSeq	CLC Genomics Workbench 7.5.1	AAZAHC000000000	SAMN17814275	GCA_017059025.1	SRR13642691
SKB114	1/2a	**1361**	940	2014	Asheville, NC, USA	N049	35.522016 N, −82.477618 W	Feces	16	2,900,601	37.8	547,356	118	844,171	Illumina MiSeq	CLC Genomics Workbench 7.5.1	AAZCQA000000000	SAMN17814345	GCA_017122785.1	SRR13642816
SKB305	1/2a	**1361**	940	2015	Asheville, NC, USA	N057	35.620224 N, −82.519569 W	Feces	28	2,863,544	37.9	321,631	880	8,882,622	Illumina NextSeq 500	FastQC v0.11.5, BBDuk v38.89	AAAXEE000000000	SAMN08183164	GCA_004622535.1	SRR6395258
SKB357	1/2a	**1361**	940	2015	Asheville, NC, USA	N014	35.618094 N, −82.497380 W	Rectal swab	18	2,991,420	37.9	505,271	332	3,444,938	Illumina MiSeq	CLC Genomics Workbench 7.5.1	AAZAQH000000000	SAMN17815144	GCA_017068715.1	SRR13643240
SKB369	1/2a	**1361**	940	2015	Asheville, NC, USA	N099	35.645548 N, −82.560473 W	Feces	14	2,877,928	37.9	547,099	78	546,293	Illumina MiSeq	CLC Genomics Workbench 7.5.1	AAZCMR000000000	SAMN17815041	GCA_017121205.1	SRR13643069
SKB430	1/2a	955	955	2016	Asheville, NC, USA	N079	35.592583 N, −82.525923 W	Feces	14	3,016,444	37.9	585,180	149	1,546,959	Illumina MiSeq	CLC Genomics Workbench 7.5.1	AAZAQI000000000	SAMN17815080	GCA_017068895.1	SRR13643181
SKB345	1/2a	956	956	2015	Asheville, NC, USA	N016	35.511962 N, −82.529787 W	Rectal swab	18	3,035,456	37.8	502,994	566	6,107,663	Illumina MiSeq	CLC Genomics Workbench 7.5.1	AAZAQJ000000000	SAMN17815085	GCA_017068475.1	SRR13643191
SKB409	1/2a	956	956	2016	Asheville, NC, USA	N108	35.590995 N, −82.537394 W	Feces	17	2,955,677	37.8	1,488,778	251	2,535,356	Illumina MiSeq	CLC Genomics Workbench 7.5.1	AAZAOW000000000	SAMN17815195	GCA_017067795.1	SRR13643293
SKB313	1/2a	983	983	2015	Asheville, NC, USA	RK CUB 91014	Unknown	Rectal swab	14	2,908,278	37.9	564,970	78	788,984	Illumina MiSeq	CLC Genomics Workbench 7.5.1	AAZAQZ000000000	SAMN17815043	GCA_017068995.1	SRR13643123
SKB57	1/2a	1055	1055	2014	Macon, GA, USA	UGA170	32.519241 N, −83.425799 W	Rectal swab	16	2,956,058	37.8	428,541	90	550,570	Illumina MiSeq	CLC Genomics Workbench 7.5.1	AAZCNR000000000	SAMN17815020	GCA_017121305.1	SRR13642986
SKB63	1/2a	**1357**	1357	2014	Asheville, NC, USA	N034	35.468036 N, −82.489578 W	Feces	53	2,883,063	37.9	115,587	28	167,582	Illumina MiSeq	CLC Genomics Workbench 7.5.1	AAZCPQ000000000	SAMN17814452	GCA_017122455.1	SRR13642935
SKB90	1/2a	**1359**	1359	2014	Macon, GA, USA	UGA177	32.547886 N, −83.521293 W	Rectal swab	14	2,976,506	37.8	528,722	79	497,075	Illumina MiSeq	CLC Genomics Workbench 7.5.1	AAZCPM000000000	SAMN17814433	GCA_017122615.1	SRR13642927
SKB113	1/2a	**1360**	1360	2014	Asheville, NC, USA	N046	35.499100 N, −82.550667 W	Feces	22	2,937,965	37.9	296,518	97	687,659	Illumina MiSeq	CLC Genomics Workbench 7.5.1	AAZCQT000000000	SAMN17814356	GCA_017122955.1	SRR13642905
SKB125	1/2a	**1364**	1364	2014	Asheville, NC, USA	N052	35.652417 N, −82.545153 W	Feces	15	2,897,294	37.9	528,714	74	518,894	Illumina MiSeq	CLC Genomics Workbench 7.5.1	AAZCRS000000000	SAMN17814132	GCA_017123775.1	SRR13642648
SKB520	1/2a	**1386**	1364	2016	Asheville, NC, USA	N124	35.566479 N, −82.589689 W	Rectal swab	29	2,862,908	37.9	423,828	779	7,828,376	Illumina NextSeq 500	FastQC v0.11.5, BBDuk v38.89	AAAXER000000000	SAMN08183146	GCA_004627135.1	SRR6396634
SKB102	1/2a	**1365**	1365	2014	Asheville, NC, USA	N041	35.618657 N, −82.377747 W	Rectal swab	12	3,067,177	37.9	542,982	101	654,669	Illumina MiSeq	CLC Genomics Workbench 7.5.1	ABJMHI000000000	SAMN31821597	GCA_026273155.1	SRR22362546
SKB128	1/2a	**1365**	1365	2014	Asheville, NC, USA	N052	35.652417 N, −82.545153 W	Feces	17	2,982,947	37.8	1,497,612	84	622,685	Illumina MiSeq	CLC Genomics Workbench 7.5.1	AAZCOT000000000	SAMN17814507	GCA_017122135.1	SRR13642959
SKB183	1/2a	**1367**	1367	2015	Asheville, NC, USA	N003	35.632554 N, −82.504740 W	Nasal swab	16	2,849,527	37.9	525,049	46	461,453	Illumina MiSeq	CLC Genomics Workbench 7.5.1	AAZAQF000000000	SAMN17815103	GCA_017068505.1	SRR13643201
SKB185	1/2a	**1367**	1367	2015	Asheville, NC, USA	N003	35.632554 N, −82.504740 W	Nasal swab	18	2,845,633	37.9	1,467,872	722	7,357,255	Illumina NextSeq 500	FastQC v0.11.5, BBDuk v38.89	AAAXDP000000000	SAMN08183179	GCA_004625115.1	SRR6392100
SKB191	1/2a	**1369**	1369	2015	Asheville, NC, USA	N006	35.634546 N, −82.499686 W	Nasal swab	25	2,875,347	37.9	321,632	829	8,680,965	Illumina NextSeq 500	FastQC v0.11.5, BBDuk v38.89	AAAXEA000000000	SAMN08183170	GCA_004624215.1	SRR6395220
SKB193	1/2a	**1369**	1369	2015	Asheville, NC, USA	N006	35.634546 N, −82.499686 W	Nasal swab	15	2,998,969	37.9	523,428	447	4,587,351	Illumina MiSeq	CLC Genomics Workbench 7.5.1	AAZAQC000000000	SAMN17815120	GCA_017068695.1	SRR13643231
SKB309	1/2a	**1369**	1369	2015	Asheville, NC, USA	N057	35.620224 N, −82.519569 W	Rectal swab	26	2,905,958	37.9	321,634	1,043	10,679,862	Illumina NextSeq 500	FastQC v0.11.5, BBDuk v38.89	AAAXEI000000000	SAMN08183163	GCA_004625565.1	SRR6395508
SKB391	1/2a	**1369**	1369	2015	Asheville, NC, USA	N057	35.620224 N, −82.519569 W	Nasal swab	16	2,995,234	37.9	523,399	251	2,612,940	Illumina MiSeq	CLC Genomics Workbench 7.5.1	AAZAPY000000000	SAMN17815136	GCA_017068795.1	SRR13643218
SKB725	1/2a	**1369**	1369	2016	Asheville, NC, USA	N150	35.653032 N, −82.489195 W	Feces	15	3,087,202	37.8	523,409	205	2,172,841	Illumina MiSeq	CLC Genomics Workbench 7.5.1	AAZAHD000000000	SAMN17814284	GCA_017059015.1	SRR13642694
SKB198	1/2a	**1371**	1371	2015	Asheville, NC, USA	N059	35.646382 N, −82.492551 W	Nasal swab	20	2,872,274	37.9	570,428	1,110	11,166,016	Illumina NextSeq 500	FastQC v0.11.5, BBDuk v38.89	AAAXDG000000000	SAMN08183188	GCA_004623375.1	SRR6391874
SKB235	1/2a	**1371**	1371	2015	Asheville, NC, USA	N072	35.600941 N, −82.537208 W	Rectal swab	19	2,870,816	37.9	570,428	1,226	12,372,196	Illumina NextSeq 500	FastQC v0.11.5, BBDuk v38.89	AAAXEU000000000	SAMN08183172	GCA_004626435.1	SRR6396903
SKB199	1/2a	**1372**	1372	2015	Asheville, NC, USA	N063	35.648012 N, −82.489036 W	Nasal swab	51	3,078,012	37.9	425,383	1,063	11,470,077	Illumina NextSeq 500	FastQC v0.11.5, BBDuk v38.89	AAAXEG000000000	SAMN08183161	GCA_004625905.1	SRR6395334
SKB265	1/2a	**1372**	1372	2015	Asheville, NC, USA	N080	35.620224 N, −82.519569 W	Rectal swab	16	2,996,697	37.9	529,306	442	4,615,515	Illumina MiSeq	CLC Genomics Workbench 7.5.1	AAZAFP000000000	SAMN17814439	GCA_017058255.1	SRR13642933
SKB266	1/2a	**1372**	1372	2015	Asheville, NC, USA	N080	35.620224 N, −82.519569 W	Rectal swab	20	2,879,254	37.9	529,167	69	700,714	Illumina MiSeq	CLC Genomics Workbench 7.5.1	AAZARE000000000	SAMN17814477	GCA_017069015.1	SRR13642936
SKB325	1/2a	**1372**	1372	2015	Asheville, NC, USA	N089	35.619101 N, −82.507734 W	Nasal swab	24	2,898,432	37.9	423,961	1,263	12,864,737	Illumina NextSeq 500	FastQC v0.11.5, BBDuk v38.89	AAAXEV000000000	SAMN08183143	GCA_004626655.1	SRR6396650
SKB428	1/2a	**1372**	1372	2016	Asheville, NC, USA	N110	35.584093 N, −82.531423 W	Rectal swab	58	2,916,174	37.9	114,851	138	1,439,357	Illumina NextSeq 500	FastQC v0.11.5, BBDuk v38.89	AAAXEC000000000	SAMN08183168	GCA_004626235.1	SRR6395235
SKB512	1/2a	**1372**	1372	2016	Asheville, NC, USA	N124	35.566479 N, −82.589689 W	Feces	24	2,899,986	37.8	329,882	1,021	10,282,021	Illumina NextSeq 500	FastQC v0.11.5, BBDuk v38.89	AAAXDR000000000	SAMN08183148	GCA_004626165.1	SRR6394746
SKB580	1/2a	**1372**	1372	2016	Asheville, NC, USA	N109	35.590995 N, −82.537394 W	Feces	18	2,905,866	37.8	527,444	53	407,517	Illumina MiSeq	CLC Genomics Workbench 7.5.1	AAZCQX000000000	SAMN17814192	GCA_017123375.1	SRR13642674
SKB274	1/2a	**1374**	1374	2015	Asheville, NC, USA	N081	35.645548 N, −82.560473 W	Rectal swab	17	2,949,710	37.9	337,428	127	890,234	Illumina MiSeq	CLC Genomics Workbench 7.5.1	AAZCQV000000000	SAMN17814269	GCA_017123235.1	SRR13642683
SKB295	1/2a	**1376**	1376	2015	Asheville, NC, USA	N085	35.645548 N, −82.560473 W	Rectal swab	27	2,857,829	37.9	291,362	1,038	10,357,963	Illumina NextSeq 500	FastQC v0.11.5, BBDuk v38.89	AAAXEB000000000	SAMN08183166	GCA_004627515.1	SRR6395254
SKB330	1/2a	**1381**	1381	2015	Asheville, NC, USA	N091	35.619101 N, −82.507734 W	Rectal swab	25	2,895,064	37.8	431,535	946	9,675,234	Illumina NextSeq 500	FastQC v0.11.5, BBDuk v38.89	AAAXDW000000000	SAMN08183177	GCA_004623995.1	SRR6394855
SKB353	1/2a	**1381**	1381	2015	Asheville, NC, USA	N094	35.619101 N, −82.507734 W	Nasal swab	25	2,935,652	37.8	356,741	70	716,619	Illumina MiSeq	CLC Genomics Workbench 7.5.1	AAZAQD000000000	SAMN17815115	GCA_017068835.1	SRR13643212
SKB545	1/2a	**1387**	1387	2016	Asheville, NC, USA	N121	35.517418 N, −82.575864 W	Rectal swab	23	2,828,993	37.9	568,823	572	5,665,666	Illumina NextSeq 500	FastQC v0.11.5, BBDuk v38.89	AAAXEN000000000	SAMN08183155	GCA_004627075.1	SRR6395801
SKB699	1/2a	**1389**	1389	2016	Asheville, NC, USA	N148	35.566479 N, −82.589689 W	Feces	20	2,963,264	37.8	459,921	86	690,705	Illumina MiSeq	CLC Genomics Workbench 7.5.1	AAZCRO000000000	SAMN17814182	GCA_017123815.1	SRR13642669
SKB149	1/2a	**1475**	1475	2014	VA, USA	VT BBRC 121	Unknown	Rectal swab	38	2,863,550	37.9	180,571	31	304,540	Illumina MiSeq	CLC Genomics Workbench 7.5.1	AAZAQG000000000	SAMN17815125	GCA_017068755.1	SRR13643233
SKB150	1/2a	**1476**	1476	2014	VA, USA	VT BBRC 122	Unknown	Feces	85	2,920,183	37.8	64,528	22	226,959	Illumina MiSeq	CLC Genomics Workbench 7.5.1	AAZAOV000000000	SAMN17815194	GCA_017067915.1	SRR13643294
SKB209	1/2a	**1477**	1477	2015	Asheville, NC, USA	N047	35.560815 N, −82.594653 W	Rectal swab	32	2,918,323	37.8	292,789	42	432,109	Illumina MiSeq	CLC Genomics Workbench 7.5.1	AAZAQT000000000	SAMN17815095	GCA_017069175.1	SRR13643196
SKB243	1/2a	**1478**	1478	2015	Asheville, NC, USA	N074	35.618079 N, −82.378587 W	Feces	14	2,966,718	37.9	524,291	565	5,896,291	Illumina MiSeq	CLC Genomics Workbench 7.5.1	AAZARQ000000000	SAMN17815036	GCA_017069535.1	SRR13643070
SKB293	1/2a	**1479**	1479	2015	Asheville, NC, USA	N085	35.645548 N, −82.560473 W	Rectal swab	17	2,850,602	37.9	421,556	64	639,119	Illumina MiSeq	CLC Genomics Workbench 7.5.1	AAZARC000000000	SAMN17815042	GCA_017069235.1	SRR13643080
SKB728	1/2a	**1483**	1483	2017	Asheville, NC, USA	N152	35.600941 N, −82.537208 W	Feces	14	3,035,504	37.8	527,573	115	1,184,669	Illumina MiSeq	CLC Genomics Workbench 7.5.1	AAZAHE000000000	SAMN17814285	GCA_017058975.1	SRR13642695
SKB231	1/2a	**1487**	1487	2015	Asheville, NC, USA	N072	35.600941 N, −82.537208 W	Feces	19	3,038,326	37.8	295,311	438	4,598,873	Illumina MiSeq	CLC Genomics Workbench 7.5.1	AAZARL000000000	SAMN17815040	GCA_017069435.1	SRR13643067
SKB701	1/2a	**1487**	1487	2016	Asheville, NC, USA	N148	35.566479 N, −82.589689 W	Rectal swab	21	3,039,759	37.8	293,870	197	2,056,553	Illumina MiSeq	CLC Genomics Workbench 7.5.1	AAZAGU000000000	SAMN17814162	GCA_017059275.1	SRR13642663
SKB269	1/2b	379	379	2015	Asheville, NC, USA	N080	35.620224 N, −82.519569 W	Nasal swab	48	2,904,573	37.9	173,236	33	339,275	Illumina MiSeq	CLC Genomics Workbench 7.5.1	AAZAQX000000000	SAMN17815079	GCA_017069035.1	SRR13643136
SKB516	1/2b	379	379	2016	Asheville, NC, USA	N124	35.566479 N, −82.589689 W	Feces	31	2,885,571	37.9	345,706	736	7,400,246	Illumina NextSeq 500	FastQC v0.11.5, BBDuk v38.89	AAAXFC000000000	SAMN08183145	GCA_004626735.1	SRR6397282
SKB781	1/2b	736	736	2017	Asheville, NC, USA	N037	35.560815 N, −82.594653 W	Nasal swab	23	3,001,405	37.9	256,949	226	2,303,773	Illumina MiSeq	CLC Genomics Workbench 7.5.1	AAZAGQ000000000	SAMN17814377	GCA_017059055.1	SRR13642911
SKB58	1/2b	**2973**	2973	2014	Macon, GA, USA	UGA170	32.519241 N, −83.425799 W	Swab	87	3,000,949	37.8	60,186	89	910,060	Illumina NextSeq 500	FastQC v0.11.5, BBDuk v38.89	DAKHOY000000000	SAMN31891309	GCA_026473625.1	SRR22428074
SKB59	1/2b	**2973**	2973	2014	Macon, GA, USA	UGA170	32.519241 N, −83.425799 W	Swab	94	3,015,184	37.8	63,864	89	911,078	Illumina NextSeq 500	FastQC v0.11.5, BBDuk v38.89	DAKHKE000000000	SAMN31891310	GCA_026470745.1	SRR22428073
SKB106	1/2c	789	789	2014	Asheville, NC, USA	N043	35.499100 N, −82.550667 W	Feces	22	2,928,853	37.8	429,378	78	572,235	Illumina MiSeq	CLC Genomics Workbench 7.5.1	AAZCLT000000000	SAMN17815138	GCA_017120565.1	SRR13643222
SKB15	1/2c	789	789	2014	Asheville, NC, USA	N023	35.619750 N, −82.582253 W	Feces	18	2,925,154	37.8	429,470	56	339,038	Illumina MiSeq	CLC Genomics Workbench 7.5.1	AAZCOK000000000	SAMN17814487	GCA_017122035.1	SRR13642967
SKB479	1/2c	**1385**	1385	2016	Asheville, NC, USA	N119	35.592583 N, −82.525923 W	Nasal swab	27	2,851,686	37.9	596,964	716	7,092,381	Illumina NextSeq 500	FastQC v0.11.5, BBDuk v38.89	AAAXEM000000000	SAMN08183150	GCA_004626915.1	SRR6396261
SKB159	4b	1	1	2015	Asheville, NC, USA	N061	35.581290 N, −82.518469 W	Feces	32	2,917,084	37.9	298,032	1,067	11,288,130	Illumina NextSeq 500	FastQC v0.11.5, BBDuk v38.89	AAAXDI000000000	SAMN08183189	GCA_004622875.1	SRR6391962
SKB350	4b	1	1	2015	Asheville, NC, USA	N094	35.619101 N, −82.507734 W	Rectal swab	201	2,966,721	37.8	41,544	98	1,005,851	Illumina MiSeq	CLC Genomics Workbench 7.5.1	AAZCLJ000000000	SAMN17815135	GCA_017120105.1	SRR13643223
SKB581	4b	1	1	2016	Asheville, NC, USA	N135	35.580980 N, −82.514457 W	Rectal swab	18	3,028,114	37.9	476,515	244	2,547,440	Illumina MiSeq	CLC Genomics Workbench 7.5.1	AAZASF000000000	SAMN17814497	GCA_017069395.1	SRR13642962
SKB682	4b	1	1	2016	Asheville, NC, USA	N132	35.620987 N, −82.523371 W	Feces	14	2,933,894	37.9	516,971	203	2,033,106	Illumina MiSeq	CLC Genomics Workbench 7.5.1	AAZAGP000000000	SAMN17814147	GCA_017059355.1	SRR13642655
SKB111	4b	1039	2	2014	Asheville, NC, USA	N044	35.618195 N, −82.378677 W	Rectal swab	19	2,975,532	37.9	329,707	75	600,860	Illumina MiSeq	CLC Genomics Workbench 7.5.1	AAZCLP000000000	SAMN17815132	GCA_017120325.1	SRR13643220
SKB112	4b	1039	2	2014	Asheville, NC, USA	N044	35.618195 N, −82.378677 W	Rectal swab	17	3,016,345	37.9	477,787	105	806,060	Illumina MiSeq	CLC Genomics Workbench 7.5.1	AAZCPV000000000	SAMN17814387	GCA_017122915.1	SRR13642910
SKB121	4b	1039	2	2014	Asheville, NC, USA	N021	35.620924 N, −82.523347 W	Feces	19	2,925,293	37.9	477,783	76	509,221	Illumina MiSeq	CLC Genomics Workbench 7.5.1	AAZCRP000000000	SAMN17814170	GCA_017123735.1	SRR13642654
SKB297	4b	1039	2	2015	Asheville, NC, USA	N085	35.645548 N, −82.560473 W	Nasal swab	30	2,900,303	37.9	307,904	870	8,920,502	Illumina NextSeq 500	FastQC v0.11.5, BBDuk v38.89	AAAXED000000000	SAMN08183165	GCA_004625335.1	SRR6395255
SKB461	4b	1039	2	2016	Asheville, NC, USA	N117	35.625263 N, −82.597632 W	Rectal swab	30	2,942,750	38.0	307,907	1,104	11,284,292	Illumina NextSeq 500	FastQC v0.11.5, BBDuk v38.89	AAAXEP000000000	SAMN08183152	GCA_004622375.1	SRR6395704
SKB537	4b	1039	2	2016	Asheville, NC, USA	N126	35.625263 N, −82.597632 W	Feces	32	2,939,836	37.9	307,899	565	5,827,138	Illumina NextSeq 500	FastQC v0.11.5, BBDuk v38.89	AAAXEW000000000	SAMN08183140	GCA_004625495.1	SRR6397048
SKB542	4b	1039	2	2016	Asheville, NC, USA	N130	35.625263 N, −82.597632 W	Feces	31	2,901,866	37.9	307,910	506	5,106,444	Illumina NextSeq 500	FastQC v0.11.5, BBDuk v38.89	AAAXEJ000000000	SAMN08183154	GCA_004627615.1	SRR6395509
SKB134	4b	631	4	2014	Asheville, NC, USA	N057	35.630684 N, −82.509354 W	Feces	14	2,903,802	38.0	593,005	78	483,920	Illumina NextSeq 500	FastQC v0.11.5, BBDuk v38.89	ABJNSY000000000	SAMN31821589	GCA_026375595.1	SRR22362539
SKB444	4b	631	4	2016	Asheville, NC, USA	N116	35.517192 N, −82.573830 W	Feces	37	2,874,823	37.9	244,554	1,342	13,828,113	Illumina NextSeq 500	FastQC v0.11.5, BBDuk v38.89	AAAXDQ000000000	SAMN08183178	GCA_004625775.1	SRR6394325
SKB467	4b	631	4	2016	Asheville, NC, USA	N061	35.517192 N, −82.573830 W	Nasal swab	24	3,003,587	37.9	251,638	152	1,556,226	Illumina MiSeq	CLC Genomics Workbench 7.5.1	AAZAQK000000000	SAMN17815082	GCA_017068875.1	SRR13643182
SKB634	4b	382	183	2016	Asheville, NC, USA	N061	35.517192 N, −82.573830 W	Nasal swab	17	3,006,694	37.9	309,733	60	481,371	Illumina MiSeq	CLC Genomics Workbench 7.5.1	AAZCRJ000000000	SAMN17814187	GCA_017123255.1	SRR13642672
SKB123	4b	388	388	2014	Asheville, NC, USA	N051	35.508438 N, −82.553691 W	Rectal swab	16	2,932,212	37.9	543,959	83	569,106	Illumina MiSeq	CLC Genomics Workbench 7.5.1	AAZCRK000000000	SAMN17814135	GCA_017123595.1	SRR13642652
SKB282	4b	388	388	2015	Asheville, NC, USA	N083	35.620224 N, −82.519569 W	Nasal swab	30	2,887,474	37.9	498,306	814	8,124,789	Illumina NextSeq 500	FastQC v0.11.5, BBDuk v38.89	AAAXEF000000000	SAMN08183160	GCA_004623635.1	SRR6395362
SKB544	4b	388	388	2016	Asheville, NC, USA	N130	35.625263 N, −82.597632 W	Feces	26	2,927,480	37.9	538,013	624	6,372,548	Illumina NextSeq 500	FastQC v0.11.5, BBDuk v38.89	AAAXDF000000000	SAMN08183186	GCA_004627595.1	SRR6391880
SKB578	4b	388	388	2016	Asheville, NC, USA	N109	35.590995 N, −82.537394 W	Feces	18	2,942,399	37.9	303,674	54	422,691	Illumina MiSeq	CLC Genomics Workbench 7.5.1	AAZCRR000000000	SAMN17814246	GCA_017123795.1	SRR13642678
SKB647	4b	388	388	2016	Asheville, NC, USA	N139	35.635396 N, −82.525301 W	Feces	20	3,008,530	37.9	538,173	174	1,789,146	Illumina MiSeq	CLC Genomics Workbench 7.5.1	AAZAHA000000000	SAMN17814124	GCA_017059325.1	SRR13642646
SKB676	4b	388	388	2016	Asheville, NC, USA	N142	35.495956 N, −82.517875 W	Rectal swab	19	3,055,291	37.9	538,212	180	1,876,916	Illumina MiSeq	CLC Genomics Workbench 7.5.1	AAZAGW000000000	SAMN17814142	GCA_017059315.1	SRR13642661
SKB698	4b	388	388	2016	Asheville, NC, USA	N148	35.566479 N, −82.589689 W	Feces	15	2,934,732	37.9	543,712	66	530,090	Illumina MiSeq	CLC Genomics Workbench 7.5.1	AAZCLK000000000	SAMN17815133	GCA_017120065.1	SRR13643213
SKB769	4b	388	388	2017	Asheville, NC, USA	N158	35.584093 N, −82.531423 W	Rectal swab	25	2,946,457	37.9	498,310	135	1,350,967	Illumina MiSeq	CLC Genomics Workbench 7.5.1	AAZAGR000000000	SAMN17814282	GCA_017059295.1	SRR13642696
SKB776	4b	388	388	2017	Asheville, NC, USA	N158	35.584093 N, −82.531423 W	Nasal swab	24	2,941,322	37.9	498,313	194	1,931,148	Illumina MiSeq	CLC Genomics Workbench 7.5.1	AAZAGS000000000	SAMN17814426	GCA_017059135.1	SRR13642922
SKB60	4b	**2145**	388	2014	Asheville, NC, USA	N034	35.468036 N, −82.489578 W	Feces	15	2,883,400	37.9	515,701	79	470,837	Illumina MiSeq	CLC Genomics Workbench 7.5.1	AAZCNJ000000000	SAMN17815029	GCA_017121425.1	SRR13643007
SKB61	4b	**2145**	388	2014	Asheville, NC, USA	N034	35.468036 N, −82.489578 W	Feces	88	2,902,099	37.9	66,824	82	812,998	Illumina NextSeq 500	FastQC v0.11.5, BBDuk v38.89	DAKHKF000000000	SAMN31891311	GCA_026470725.1	SRR22428072
SKB103	4b	554	554	2014	Asheville, NC, USA	N042	35.508438 N, −82.553691 W	Feces	14	2,913,078	37.9	408,295	83	598,058	Illumina MiSeq	CLC Genomics Workbench 7.5.1	AAZCQY000000000	SAMN17814186	GCA_017123295.1	SRR13642673
SKB104	4b	554	554	2014	Asheville, NC, USA	N042	35.508438 N, −82.553691 W	Rectal swab	16	2,947,680	37.9	408,295	108	787,268	Illumina MiSeq	CLC Genomics Workbench 7.5.1	AAZCKY000000000	SAMN17815142	GCA_017120265.1	SRR13643242
SKB155	4b	554	554	2015	Asheville, NC, USA	N060	35.581291 N, −82.518469 W	Rectal swab	34	2,911,678	37.9	307,794	1,000	10,851,154	Illumina NextSeq 500	FastQC v0.11.5, BBDuk v38.89	AAAXDV000000000	SAMN08183187	GCA_004624655.1	SRR6394375
SKB361	4b	554	554	2015	Asheville, NC, USA	N095	35.517192 N, −82.573830 W	Rectal swab	21	3,029,841	37.9	408,295	181	1,907,733	Illumina MiSeq	CLC Genomics Workbench 7.5.1	AAZCQU000000000	SAMN17814247	GCA_017123135.1	SRR13642680
SKB373	4b	554	554	2015	Asheville, NC, USA	N099	35.645548 N, −82.560473 W	Feces	18	2,987,566	37.9	408,295	208	2,161,193	Illumina MiSeq	CLC Genomics Workbench 7.5.1	AAZCLX000000000	SAMN17815086	GCA_017120615.1	SRR13643192
SKB379	4b	554	554	2015	VA, USA	VT BBRC 119	Unknown	Feces	33	2,892,284	37.9	308,144	929	10,067,487	Illumina NextSeq 500	FastQC v0.11.5, BBDuk v38.89	AAAXDX000000000	SAMN08183174	GCA_004626315.1	SRR6394911
SKB441	4b	554	554	2016	Asheville, NC, USA	N092	35.491520 N, −82.564499 W	Feces	32	2,899,903	37.9	307,794	1,206	12,519,329	Illumina NextSeq 500	FastQC v0.11.5, BBDuk v38.89	AAAXDT000000000	SAMN08183167	GCA_004624955.1	SRR6394748
SKB494	4b	554	554	2016	Asheville, NC, USA	N123	35.648864 N, −82.479895 W	Feces	30	2,955,636	37.8	303,515	1,267	13,087,194	Illumina NextSeq 500	FastQC v0.11.5, BBDuk v38.89	AAAXDS000000000	SAMN08183149	GCA_004623955.1	SRR6394738
SKB513	4b	554	554	2016	Asheville, NC, USA	N124	35.566479 N, −82.589689 W	Feces	30	2,889,359	37.9	308,144	1,007	10,132,309	Illumina NextSeq 500	FastQC v0.11.5, BBDuk v38.89	AAAXEQ000000000	SAMN08183147	GCA_004624395.1	SRR6396513
SKB536	4b	554	554	2016	Asheville, NC, USA	N126	35.625263 N, −82.597632 W	Feces	30	2,959,542	37.9	295,426	870	9,131,000	Illumina NextSeq 500	FastQC v0.11.5, BBDuk v38.89	AAAXDO000000000	SAMN08183183	GCA_004625675.1	SRR6392040
SKB543	4b	554	554	2016	Asheville, NC, USA	N130	35.625263 N, −82.597632 W	Feces	28	2,902,028	37.9	303,515	795	8,056,112	Illumina NextSeq 500	FastQC v0.11.5, BBDuk v38.89	AAAXFD000000000	SAMN08183144	GCA_004627115.1	SRR6397911
SKB558	4b	554	554	2016	Asheville, NC, USA	N131	35.648864 N, −82.479895 W	Feces	27	2,957,295	37.8	308,144	691	7,103,431	Illumina NextSeq 500	FastQC v0.11.5, BBDuk v38.89	AAAXET000000000	SAMN08183185	GCA_004624435.1	SRR6396649
SKB614	4b	554	554	2016	Asheville, NC, USA	N087	35.603350 N, −82.527849 W	Rectal swab	18	2,933,532	37.9	408,294	65	510,190	Illumina MiSeq	CLC Genomics Workbench 7.5.1	AAZCRD000000000	SAMN17814196	GCA_017123315.1	SRR13642675
SKB757	4b	554	554	2017	Asheville, NC, USA	N157	35.570732 N, −82.499853 W	Feces	23	2,943,198	37.9	296,833	123	1,228,096	Illumina MiSeq	CLC Genomics Workbench 7.5.1	AAZCQM000000000	SAMN17814281	GCA_017123215.1	SRR13642692
SKB96	4b	554	554	2014	Asheville, NC, USA	N039	35.564102 N, −82.489931 W	Feces	12	2,915,645	37.9	540,987	70	438,738	Illumina MiSeq	CLC Genomics Workbench 7.5.1	AAZCPX000000000	SAMN17814398	GCA_017122875.1	SRR13642912
SKB97	4b	554	554	2014	Asheville, NC, USA	N039	35.564102 N, −82.489931 W	Feces	14	2,987,892	37.9	540,897	96	606,506	Illumina MiSeq	CLC Genomics Workbench 7.5.1	AAZCPS000000000	SAMN17814418	GCA_017122675.1	SRR13642918
SKB98	4b	554	554	2014	Asheville, NC, USA	N039	35.564102 N, −82.489931 W	Rectal swab	15	2,992,035	37.9	408,295	114	822,705	Illumina MiSeq	CLC Genomics Workbench 7.5.1	AAZCQW000000000	SAMN17814249	GCA_017123455.1	SRR13642679
SKB317	4b	999	554	2015	Asheville, NC, USA	RK5615	35.566807 N, −82.584675 W	Rectal swab	27	2,889,958	37.9	308,152	712	7,127,335	Illumina NextSeq 500	FastQC v0.11.5, BBDuk v38.89	AAAXES000000000	SAMN08183156	GCA_004626675.1	SRR6395721
SKB42	4b	999	554	2014	NC, USA	UNK 1	Unknown	Feces	17	2,935,295	37.9	408,287	87	537,195	Illumina MiSeq	CLC Genomics Workbench 7.5.1	AAZCNZ000000000	SAMN17815017	GCA_017121285.1	SRR13642978
SKB43	4b	999	554	2014	Asheville, NC, USA	N031	35.580511 N, −82.498622 W	Swab	70	2,895,361	37.9	85,377	58	579,252	Illumina NextSeq 500	FastQC v0.11.5, BBDuk v38.89	DAKHKD000000000	SAMN31891307	GCA_026470685.1	SRR22428076
SKB45	4b	999	554	2014	Asheville, NC, USA	N031	35.580511 N, −82.498622 W	Rectal swab	15	2,910,935	37.9	408,287	74	443,013	Illumina MiSeq	CLC Genomics Workbench 7.5.1	AAZCPO000000000	SAMN17814438	GCA_017122435.1	SRR13642934
SKB46	4b	999	554	2014	Asheville, NC, USA	N031	35.580511 N, −82.498622 W	Swab	90	2,907,630	37.9	62,497	82	808,884	Illumina NextSeq 500	FastQC v0.11.5, BBDuk v38.89	DAKHMB000000000	SAMN31891308	GCA_026471725.1	SRR22428075
SKB275	4b	**1375**	554	2015	Asheville, NC, USA	N081	35.645548 N, −82.560473 W	Rectal swab	19	3,073,987	37.9	408,354	126	910,517	Illumina MiSeq	CLC Genomics Workbench 7.5.1	AAZCQZ000000000	SAMN17814271	GCA_017123465.1	SRR13642682
SKB321	4b	**1378**	554	2015	Asheville, NC, USA	N089	35.619101 N, −82.507734 W	Feces	31	2,900,741	37.9	307,904	844	8,601,599	Illumina NextSeq 500	FastQC v0.11.5, BBDuk v38.89	AAAXFA000000000	SAMN08183142	GCA_004627255.1	SRR6399033
SKB329	4b	**1378**	554	2015	Asheville, NC, USA	N091	35.619101 N, −82.507734 W	Rectal swab	25	2,902,558	37.9	307,919	1,075	10,918,862	Illumina NextSeq 500	FastQC v0.11.5, BBDuk v38.89	AAAXEH000000000	SAMN08183159	GCA_004623535.1	SRR6395462
SKB337	4b	**1378**	554	2015	Asheville, NC, USA	N093	35.619101 N, −82.507734 W	Rectal swab	30	2,901,161	37.9	303,526	881	8,951,458	Illumina NextSeq 500	FastQC v0.11.5, BBDuk v38.89	AAAXEY000000000	SAMN08183157	GCA_004624275.1	SRR6397297
SKB341	4b	**1378**	554	2015	Asheville, NC, USA	N093	35.619101 N, −82.507734 W	Nasal swab	20	3,029,015	37.9	303,526	377	3,880,975	Illumina MiSeq	CLC Genomics Workbench 7.5.1	AAZCMI000000000	SAMN17815092	GCA_017120525.1	SRR13643199
SKB349	4b	**1378**	554	2015	Asheville, NC, USA	N094	35.619101 N, −82.507734 W	Rectal swab	25	2,911,540	37.9	327,771	589	5,931,561	Illumina MiSeq	CLC Genomics Workbench 7.5.1	AAZCLI000000000	SAMN17815134	GCA_017120385.1	SRR13643211
SKB374	4b	801	651	2015	Asheville, NC, USA	UNK CUB 9415	35.575050 N, −82.631075 W	Feces	20	3,053,097	37.9	498,503	236	2,561,831	Illumina MiSeq	CLC Genomics Workbench 7.5.1	AAZCMB000000000	SAMN17815087	GCA_017120645.1	SRR13643189
SKB770	4b	801	651	2017	Asheville, NC, USA	N158	35.584093 N, −82.531423 W	Rectal swab	17	3,041,941	37.9	544,009	125	1,300,339	Illumina MiSeq	CLC Genomics Workbench 7.5.1	AAZCPU000000000	SAMN17814423	GCA_017122895.1	SRR13642915
SKB775	4b	801	651	2017	Asheville, NC, USA	N158	35.584093 N, −82.531423 W	Nasal swab	18	2,980,467	37.9	498,505	141	1,432,594	Illumina MiSeq	CLC Genomics Workbench 7.5.1	AAZCPP000000000	SAMN17814424	GCA_017122635.1	SRR13642920
SKB146	Lineage III	264	264	2014	VA, USA	VT BBRC 121	Unknown	Feces	25	3,030,315	37.9	386,361	841	9,160,449	Illumina NextSeq 500	FastQC v0.11.5, BBDuk v38.89	AAAXDL000000000	SAMN08183181	GCA_004624105.1	SRR6392089
SKB142	Lineage III	**1366**	434	2014	VA, USA	VT BBRC 119	Unknown	Feces	14	2,960,623	38.1	489,760	117	851,785	Illumina MiSeq	CLC Genomics Workbench 7.5.1	AAZCQE000000000	SAMN17814322	GCA_017123115.1	SRR13642765
SKB75	Lineage III	**1358**	1358	2014	Macon, GA, USA	UGA175	32.466313 N, −83.576571 W	Rectal swab	26	2,906,536	38.0	234,666	72	439,480	Illumina MiSeq	CLC Genomics Workbench 7.5.1	AAZCPG000000000	SAMN17814432	GCA_017122535.1	SRR13642928
SKB118	Lineage III	**1363**	1363	2014	Macon, GA, USA	UGA179	32.548795 N, −83.452115 W	Rectal swab	20	2,974,737	37.8	541,748	71	456,905	Illumina MiSeq	CLC Genomics Workbench 7.5.1	AAZCQC000000000	SAMN17814346	GCA_017122775.1	SRR13642815
SKB119	Lineage III	**1363**	1363	2014	Macon, GA, USA	UGA179	32.548795 N, −83.452115 W	Rectal swab	23	2,985,300	37.8	546,918	68	483,223	Illumina MiSeq	CLC Genomics Workbench 7.5.1	AAZCRM000000000	SAMN17814157	GCA_017123555.1	SRR13642657
SKB259	Lineage III	**1373**	1373	2015	Asheville, NC, USA	N079	35.620224 N, −82.519569 W	Nasal swab	13	2,934,131	38.0	527,500	119	833,950	Illumina MiSeq	CLC Genomics Workbench 7.5.1	AAZCQO000000000	SAMN17814273	GCA_017123175.1	SRR13642688
SKB381	Lineage III	**1382**	1382	2015	VA, USA	VT BBRC 127	Unknown	Feces	13	2,959,058	38.0	588,980	54	405,818	Illumina MiSeq	CLC Genomics Workbench 7.5.1	AAZCRC000000000	SAMN17814248	GCA_017123335.1	SRR13642677
SKB782	Lineage III	**1486**	1486	2017	Asheville, NC, USA	N037	35.560815 N, −82.594653 W	Nasal swab	25	3,119,431	37.8	522,775	171	1,821,171	Illumina MiSeq	CLC Genomics Workbench 7.5.1	AAZCPL000000000	SAMN17814430	GCA_017122575.1	SRR13642923
SKB67	Lineage III	**2158**	2158	2016	Macon, GA, USA	UGA174	32.481646 N, −83.578157 W	Rectal swab	12	2,996,511	37.9	596,909	90	559,546	Illumina MiSeq	CLC Genomics Workbench 7.5.1	AAZCPH000000000	SAMN17814467	GCA_017122495.1	SRR13642931

a Sequence type (ST) designations in bold are novel STs first identified in this study.

Fecal samples and rectal and nasal swabs were enriched for *Listeria* using the ISO method with a Half Fraser primary enrichment at 30°C for 24 to 48 h followed by a Full Fraser secondary enrichment at 37°C for 48 h, as previously described (4). Strains were streaked from frozen stocks (−80°C) on tryptic soy (TS) agar (Becton, Dickinson & Co.) and incubated at 37°C overnight. Genomic DNA was extracted by the DNeasy blood and tissue kit (Qiagen, Valencia, CA) from strains grown overnight at 37°C in brain heart infusion or TS broth. Libraries were prepared using 0.5 to 1 ng of genomic DNA with a Nextera XT DNA library preparation kit (Illumina, San Diego, CA, USA). Genomes were sequenced using either a NextSeq 500 sequencer with the NextSeq 500/550 high-output kit v2.5 (300 cycles, 2 by 150 bp) (Illumina) or a MiSeq desktop sequencer with the MiSeq kit v2 (500 cycles, 2 by 250 bp) (Illumina) according to the manufacturer’s instructions (Table 1). Raw sequencing reads were quality controlled by FastQC v0.11.5 (7) and trimmed using BBDuk2 from BBTools v38.89 (https://sourceforge.net/projects/bbmap/) or using CLC Genomics Workbench 7.5.1 (CLC Bio, Boston, MA) (Table 1). Reads were deposited into the National Center for Biotechnology Information (https://www.ncbi.nlm.nih.gov), and assemblies produced by SKESA v 2.2 (8) were annotated using the NCBI Prokaryotic Genome Annotation Pipeline (PGAP) v 6.3 (9). STs and CCs were identified using BIGSdb-Lm, hosted by the Institut Pasteur (https://bigsdb.pasteur.fr/listeria/) (10). Default parameters were used for all software.

### Data availability.

This whole-genome shotgun project has been deposited in GenBank under the accession numbers found in Table 1. The versions described in this paper are the first version.
